# Supplementary material for: Learning from imagined experiences via an endogenous prediction error
Source: Nat Commun. 2025 Dec 10;16:10845. doi: 10.1038/s41467-025-66396-2 (PMC12696094; doi:10.1038/s41467-025-66396-2)
Supplement: Supplementary file 1 — Supplementary Information [file 41467_2025_66396_MOESM1_ESM.pdf]

Learning from imagined experiences via an endogenous prediction error

Aroma Dabas, Rasmus Bruckner, Heidrun Schultz, Frederik Bergmann, Roland G. Benoit

Supplementary Information

Supplementary Methods

1. Scenario selection

We created a corpus of 129 sentences describing 67 pleasant and 62 neutral-to-unpleasant scenarios. We validated this categorization in a separate sample of participants (n = 107; 57 female, 50 male; age: M = 24.1 y; SD = 3.8 y, range = 19 y to 35 y). Similar to our main experiment, we first requested the participants to provide names of people that they were personally familiar with, and then identified four individuals who they felt neutral towards. Participants were then presented with a scenario and a given individual. They were instructed to immediately immerse themselves in the scenario and to imagine this scenario together with the respective person as vividly as possible. After simulating the interaction for 8 s, participants were prompted to assess the pleasantness of the interaction within 5 s using a continuous scale ranging from "very unpleasant" to "very pleasant," with "neutral" positioned at the midpoint.

We then compared pleasantness ratings between the two scenario conditions while controlling for participant variability using a linear mixed-effects model. Our analysis corroborated that the scenarios categorized as pleasant elicited significantly higher pleasantness ratings compared to the scenarios categorizes as neutral-to-unpleasant ($B = 0.37$, $SE = 4.5e\text{-}03$, $z = 82.48$, $p < 0.001$; Fig. S1a). We further confirmed that this was also the case in the main study ($B = 0.46$, $SE = 5.0e\text{-}03$, $z = 91.48$, $p < 0.001$; Fig. S1b).

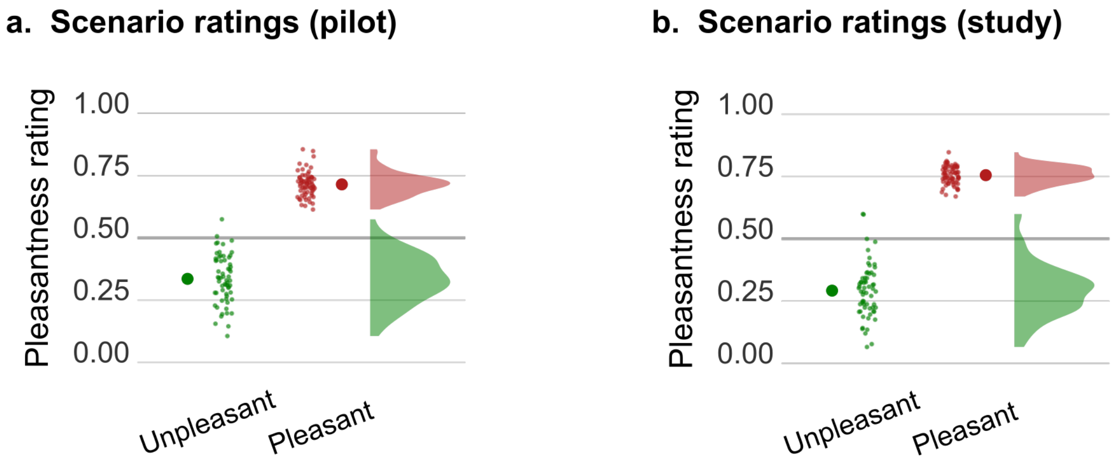

Figure S1. Scenario ratings from **a,** pilot study (n = 107) and **b,** main study (n = 49). Smaller dots denote mean ratings for each scenario. Larger dots denote mean ratings for the conditions.

2. Computational models

i. Model description

Our key model of interest, i.e. the Rescorla-Wagner (RW) model, has been described in the methods section of the main text. Below, we outline the details of the four additional models that capture different decision-making policies.

a) Choice Kernel (CK) model

The CK model captures behavior that merely repeats previous choices. Specifically, this model tracks the frequency with which all choices were previously selected, in the form of a 'choice kernel', and uses the information to predict choice repetition. The model does not take into account the reward experienced during the simulation. It is implemented as follows:

$$CK_{t+1}^{k} = CK_{t}^{k} + \alpha_c(a_t^k - CK_t^k)$$

(S1)

where CK_t^k denotes the choice kernel on trial t and for option k. α_c refers to the choice kernel learning rate and a_t^k denotes choice repetition such that a_t^k = 1 if option k is selected on trial t, otherwise a_t^k = 0. The value of α_c ranges from 0 to 1, controlling the degree to which the choice repetition (or switch) updates the kernel. We assume that the initial value of the choice kernel for all options is 0. To convert the kernel into choice probabilities, we use the softmax decision rule:

$$p_t^k = \frac{\exp(\beta_c CK_t^k)}{\sum_{i=1}^{K} \exp(\beta_c CK_t^i)}$$

(S2)

where β corresponds to the inverse temperature associated with the choice kernel. Overall, this model has the two free parameters α_c and β_c.

b) Combined Rescorla-Wagner and Choice Kernel (RW-CK) model

The combined Rescorla-Wagner and choice kernel (RW-CK) model captures choice behavior based on both the value update (which updates according to equation 1 in the main manuscript) and the choice kernel (which updates as per equation S1 in this document). The choice probability is computed by combining the outputs of the RW model and the CK model as follows:

$$p_t^k = \frac{\exp(\beta Q_t^k + \beta_c CK_t^k)}{\sum_{i=1}^{K} \exp(\beta Q_t^i + \beta_c CK_t^i)}$$

(S3)

where β and β_c denote the inverse temperature parameter associated with choice value Q_t^i and choice kernel CK_t^i, respectively. In addition to the inverse temperature parameters, this model has two more free parameters: The learning rates α (Eqn. 1) and α_c (Eqn. S1) of choice value and kernel, respectively.

c) Win-Stay-Lose-Shift (WSLS) model

This model adapts its behavior according to previous feedback, favoring actions that have been rewarded and avoiding those that haven't. This strategy, known as the win-stay lose-shift rule, is applied with a probability of 1 - $\epsilon/2$, where $\epsilon/2$ captures choice stochasticity. The total probability of staying with option k at time t is thus the combination of two cases: staying after a win with probability 1- $\epsilon/2$ and staying after a loss with probability $\epsilon/2$:

$$p_t^k = \begin{cases} 1-\epsilon/2 & \text{if } (c_{t-1} = k \text{ and } r_{t-1} = 1) \text{ or } (c_{t-1} \neq k \text{ and } r_{t-1} = 0) \\ \epsilon/2 & \text{if } (c_{t-1} \neq k \text{ and } r_{t-1} = 1) \text{ or } (c_{t-1} = k \text{ and } r_{t-1} = 0) \end{cases}$$

(S4)

In the WSLS model, ϵ is the only free parameter.

d) Null model

This model captures random choice behavior. The probability of selecting one of the two presented options is fixed at 0.5. This model thus has no free parameter.

ii. Parameter recovery

Before collecting data, we tested whether our fitting procedure can return meaningful parameter values. We followed the approach outlined by Wilson & Collins (2019)[1]. We first simulated data with known parameter values and then estimated the parameters that best describe the simulated data. In an ideal situation, the estimation procedure should yield the parameter value that was used for generating the data.

We ran the fitting procedure 50 times with different random initial conditions to increase the likelihood of finding global minima. Post data collection, we updated the parameter space of the models to match the range recovered from fitting participants' data (Table S1). The difference between the true and estimated parameter values is plotted in Fig. S2.

Table S1: Parameter space of the models

**Model**	**Parameter**	**Lower threshold**	**Upper threshold**
Win-stay lose-shift	Epsilon	0	1
Rescorla-Wagner (RW)	Alpha	0.05	1
	Beta	0	25
Choice Kernel (CK)	Alpha	0.05	1
	Beta	0	25

Note. The parameter space for the combined RW-CK model comprised the space from the individual RW and CK models.

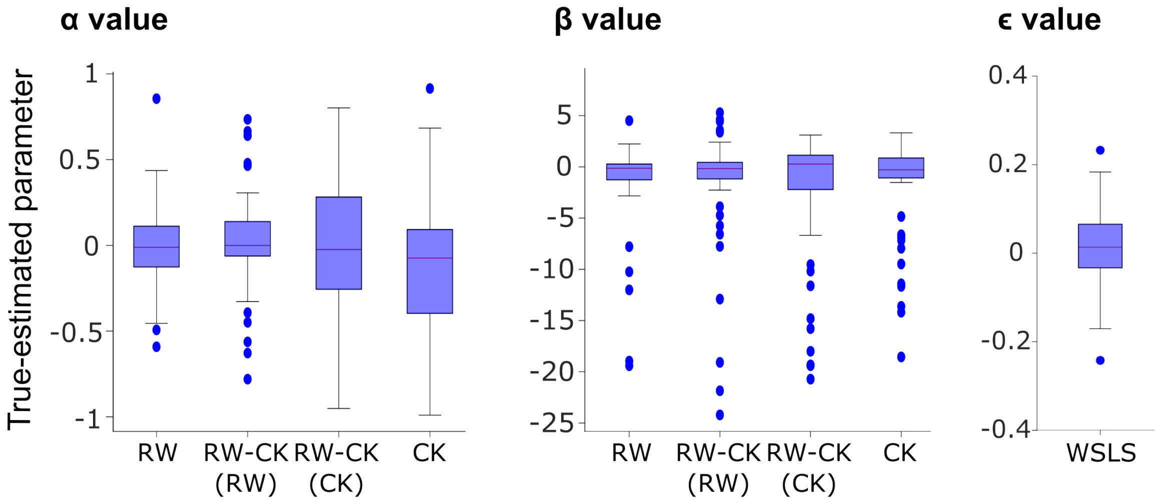

Figure S2. The difference in the estimated parameter value from its true parameter value. The median of the boxplots lies close to the 0 value indicating that overall the fitting procedure estimates parameters accurately and without a systematic estimation bias. However, the variability of the estimated parameters is considerably larger for the CK components compared to the RW components. The recovery is based on 50 simulated data sets. The whiskers extend to 1.5 x interquartile range. RW: Rescorla-Wagner model; RW-CK (RW): Rescorla-Wagner part of the combined Rescorla-Wagner Choice-Kernel model; RW-CK (CK): Choice-Kernel part of the RW-CK model; CK: Choice-Kernel; WSLS: Win-Stay-Lose-Shift model.

iii. Model recovery

We also tested whether our fitting procedure can arbitrate different models. First, we tested whether the model that generates the data is also the model that best fits the data. We illustrate this with a confusion matrix (Fig. S3a and S3b). A confusion matrix calculates the probability that the data generated by one model is best fit by a given model, i.e. *p*(fit model = B l simulated model = A), considering all models within the model space. A perfect model recovery would result in an identity matrix. The confusion matrix estimated using the Bayesian Information Criterion (BIC) values suggest that the data generated by WSLS, RW, and CK models are best recovered by

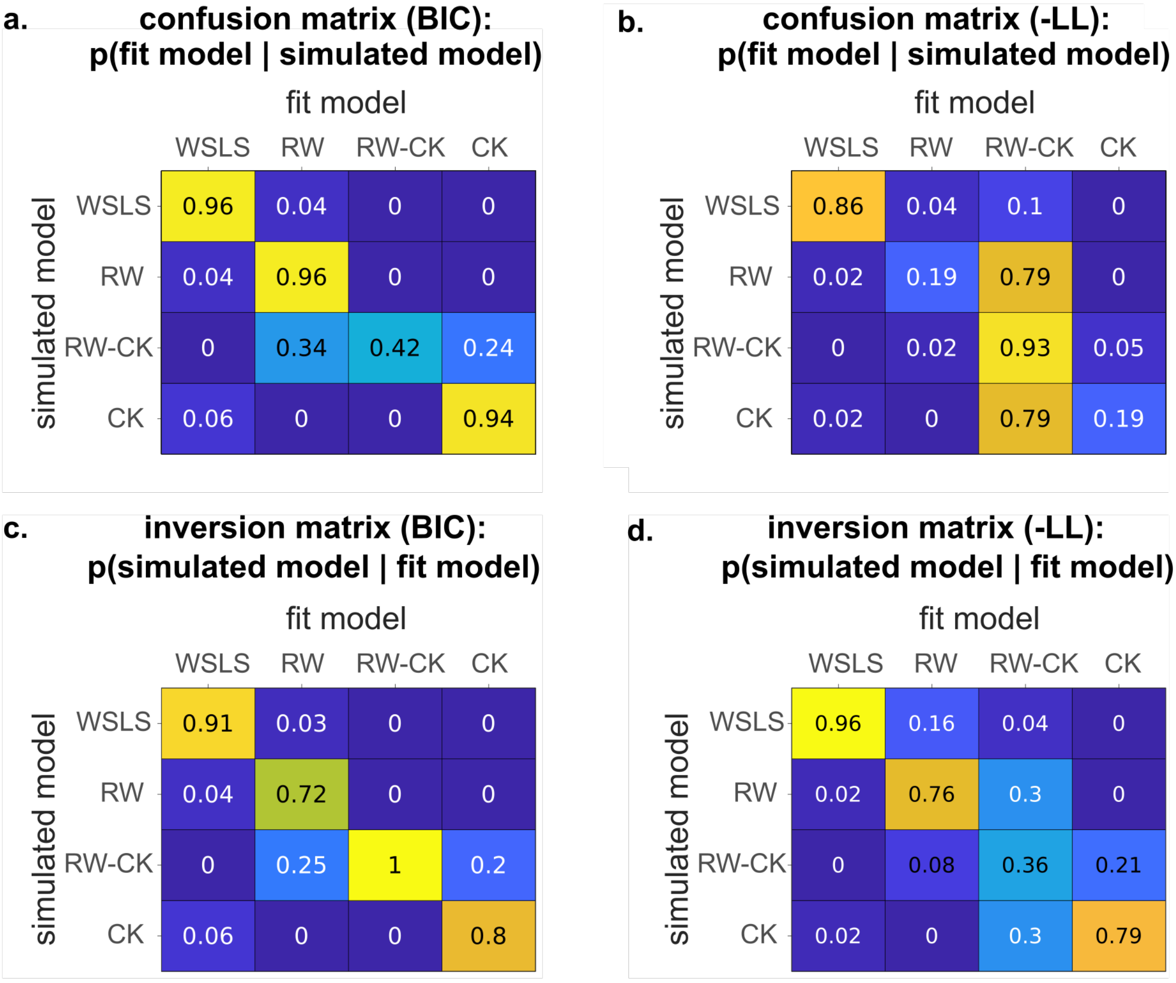

Figure S3. Model recovery confusion matrices (CM) and inversion matrices (IM). **a,** As indicated by a CM derived from BIC values, the data from WSLS, RW, and CK models can be accurately recovered. The combined RW and CK model (RW-CK) shows moderate performance in recovering its ground-truth model, with a portion of the data being better explained by the simple RW (34%) and CK (24%) models. **b**, We tested if RW-CK can best recover its own data when it is not being penalized for its additional free parameters. Minimizing the negative log-likelihood, we find that the RW-CK model recovery improves drastically (93%). As expected, it also captures the data generated by the simple RW (79%) and CK (79%) models. **c,** Additionally, we computed IM based on the CM obtained from BIC (subplot a). The matrix indicates that though the recovery of RW-CK was moderate (42%), we can be certain that it generated the data, given that it fits the data best (100%). **d**, In contrast, our confidence that RW-CK model generated its data (given that it fits the data best) drops to 36% when estimating model recovery using log-likelihood values.

their own model (Fig. S3a). Though the RW-CK model moderately recovered its own data, the remaining data generated by this model, however, were recovered by the simple RW and CK models. This observation may reflect the shared features of the RW-CK model and the individual RW and CK models. Additionally, considering that BIC was employed to compute the confusion matrix, the model recovery of RW-CK might have been impacted by the penalization of the additional two free parameters.

We thus assessed whether the RW-CK model recovery improves by using log-likelihood values instead of BIC values. Indeed, the RW-CK model successfully recovered its own data as well as the data generated by the RW and CK models (Fig. S3b).

As suggested by Wilson & Collins (2019)[1], we also examined the inverse of the confusion matrix, i.e., *p*(simulated model | fit model). Simply put, the matrix allows us to make the following inference: Given that a model fits data, which model likely generated the data? This inversion matrix offers better interpretability when the true model is unknown, which is often the case when testing the fit of a model to participant data. The matrix is computed from the confusion matrix by Bayes rule and referred to as inversion matrix. The inversion matrix computed with BIC (Fig. S3c) shows a high confidence (100%) that the RW-CK model generated the data given that it fits the data best. By contrast, when using log-likelihood values, the probability is drastically reduced that the RW-CK generated its own data, given that it fits the data best. Consequently, we used the BIC values for analyzing the model fit of the empirical data.

Supplementary Note 1

RW-estimated HR probability correlates with simulation-based liking update

To assess the overall probability of the RW model to select the HR people, we first estimated trial-wise probabilities of selecting a HR person. This was done using the RW-based choice values and the softmax rule (see Eqn. 2). We then computed the area under this curve (AUC) using the approach outlined by Pruessner et al. (2003)[2]. Across participants, the AUC correlated with the increase in liking for the HR people as measured on the external rating task (robust skipped Spearman's correlation between AUC and HR relative to LR update: r_s = .46, 95% CI = [.20 .66]; robust skipped Spearman's correlation between AUC and HR update: r_s = .41, 95% CI = [.16 .60]; Fig. S4).

A greater RW-estimated probability of selecting HR people correlates with a greater liking update

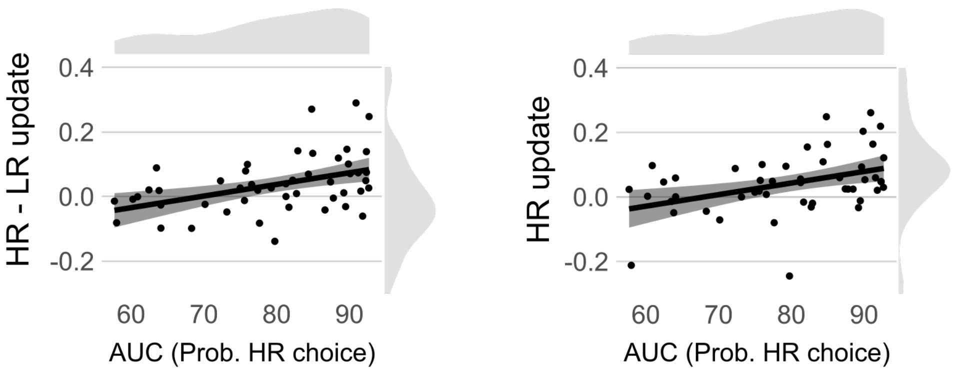

Figure S4. The RW model estimation of HR probability correlated with **a**, the update in liking for HR vs LR people **b**, and also with the update in liking for HR people without adjusting for the update for LR people. n = 49 participants.

Supplementary Note 2

Episodic simulation induces change in familiarity

We also assessed whether repeated simulations change the perceived familiarity of the imagined persons. Participants therefore rated the familiarity of each HR and LR person before and after the simulation task. They also rated two people who were not part of the task, serving as a baseline condition. To quantify a change in familiarity, we subtracted the pre-task familiarity ratings from the post-task ratings. We then corrected the change scores for the HR and LR conditions by subtracting those of the baseline condition.

We observed an increase in familiarity for both HR ($t(48) = 2.35$, $p = .01$, $d = .33$, 95% CI = [0.01 Inf]) and LR ($W = 788$, $p = .04$, $r = 0.25$, 95% CI = [0.001 Inf]; Shapiro Wilk $W = .92$, $p = .002$) people (Fig. S5). Notably, the difference between the HR and LR conditions was not significant ($t(48) = 1.36$, $p = .18$, $d = .19$, 95% CI = [-0.01 0.06]), unlike for the liking ratings.

This finding aligns with previous research showing that repeated simulations can enhance the perceived plausibility or likelihood of experiencing an event with a particular person [3,4].

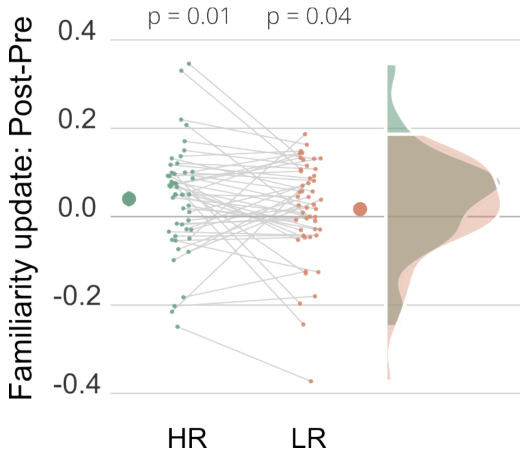

Figure S5. Significant increase in familiarity for both HR and LR people after episodic simulation. The small dots represent results from each participant (n = 49) connected by a grey line. The big dots represent the mean familiarity update for each condition.

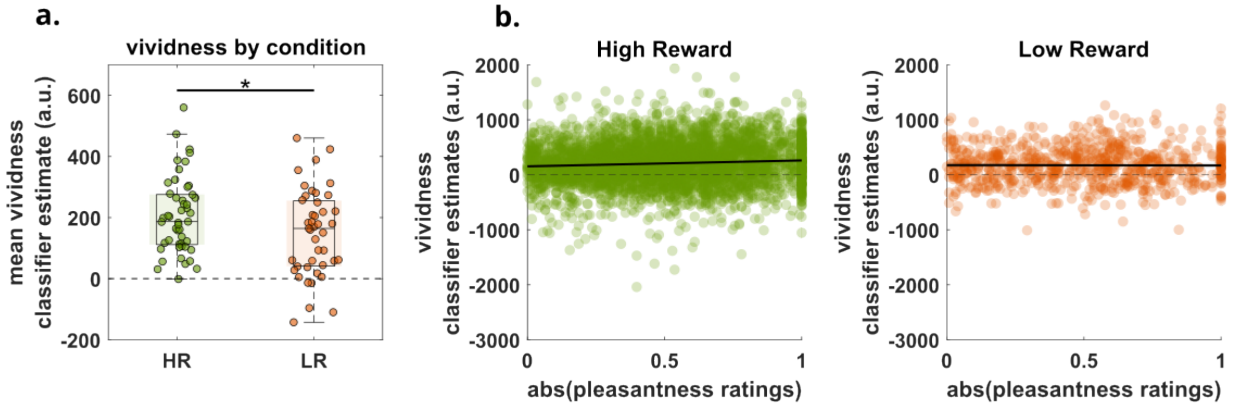

Figure S6. a, A neural signature of the vividness of prospective thought[5] yielded positive vividness scores for both conditions. Notably, the decoded vividness was greater in the HR condition which also led to a stronger value update ($t(42) = 3.22$, $p = .001$, $d = .49$). **b,** A linear mixed model with the fixed effects of decoded vividness and condition (HR vs LR) as well as the random intercept for participant predicted the absolute pleasantness value on a trial-by-trial basis. This analysis yielded a significant interaction between the two fixed factors ($t(4605) = 3.63$; $p < 0.001$). Separate follow-up models revealed that the interaction reflected a positive effect of decoded vividness in the HR ($t(3846) = 3.59$, $p < 0.001$) though not in the LR condition ($t(759) = -1.57$, $p = .12$). A greater vividness was thus associated, on a trial-by-trial basis, with a stronger experienced affect in the high reward condition that also led to a stronger value update. These analyses further corroborate that learning occurred as a consequence of the internal simulation of vivid experiences.

Supplementary Note 3

Transient or sustained modulation of striatal activity?

We performed Bayesian Model Comparison (BMC) of two model variants. Model 1 (“boxcar model”) is the model underlying our ventral striatum results in Figure 3a and described in the Methods section. Model 2 (“impulse model”) is identical to model 1, with the exemption that the simulation period and, by extension, its parametric modulation by PE is modelled with an event duration of 0s, rather than the entire length of the simulation period (8s).

First, we computed whole-brain BIC maps for each participant and model using the MACS toolbox [6]. Next, we computed average BIC values for each participant and model across the mask of the ventral striatum. Finally, we performed BMC on the subject- and model-specific BIC values via the VBA toolbox [7] (Table S2). This analysis indicates that, on a group level as well as in the majority of individual participants, the boxcar model outperforms the impulse model. We thus conclude that the ventral-striatum response in Fig. 3a more likely reflects a sustained effect as a consequence of the evaluation of the unfolding mental simulation.

Table S2: fMRI model comparison

Model	BIC	Number favoring	Exceedance probability	Model frequency
**boxcar**	3289.57 ± 42.5<br>161189	33/49	>0.999	0.736
**impulse**	3291.58 ± 42.2<br>161287	16/49	<0.001	0.264

Note. Shown for each model: Bayesian Information Criteria (BIC) averaged across each participant’s ventral-striatum mask, denoted are mean ± standard error of the mean as well as sum over participants; the number of subjects favoring each model based on BIC scores; exceedance probability and model frequency from the Bayesian Model Comparison.

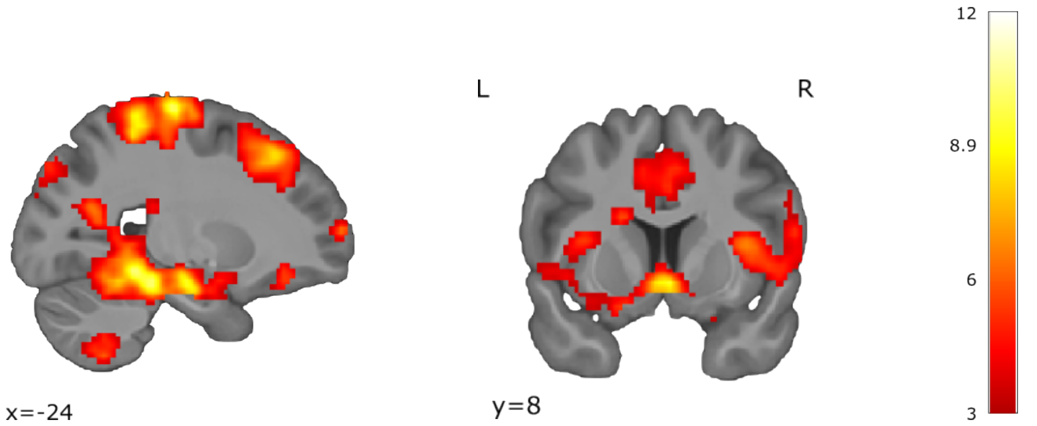

Figure S7. Prediction-error parametric modulation analysis (n = 49). The sagittal slice illustrates the cluster encompassing the hippocampus and parahippocampal cortex. The coronal slice shows the cluster in the bilateral ventral striatum, specifically the nucleus accumbens. For illustration purposes, the *t*-map was thresholded at $p < .001$ (uncorrected) with a minimum cluster size of 20 voxels and overlaid on the mean T1 image.

Table S3. Prediction error modulates univariate whole-brain activity.

AAL Label	Hemisphere	Voxels	x	y	z	*t*-value
Olfactory [1]	L	11223	-4	22	-10	12.22
Fusiform [2]	L		-27	-33	-21	11.44
Olfactory [1]	R		1	12	-7	11.07
Precuneus [4]	L		-7	-58	18	10.97
ParaHippocampal [2,3]	L		-19	-13	-21	10.94
Fusiform [2,3]	R		28	-38	-15	10.36
Frontal_Med_Orb [5]	L		-9	47	-12	9.86
Postcentral	L	8810	-39	-30	62	12.18
Precentral	L		-27	-23	70	10.85
Postcentral	L		-27	-40	67	9.44
Frontal_Mid	L	451	-24	29	48	8.52
Frontal_Sup	L		-22	34	34	5.67
Cerebelum_8	L	517	-22	-58	-56	6.67
			-19	-60	-46	6.29
Cerebelum_7b	L		-12	-75	-48	6.07
Caudate	L	46	-17	9	23	5.88
Frontal_Inf_Orb	L	77	-29	34	-12	5.79
Temporal_Inf	L	52	-54	-48	-15	5.15
Cerebelum_Crus2	R	28	48	-68	-40	4.83
Frontal_Inf_Orb	R	27	28	37	-12	4.42
Frontal_Sup_Orb	R		18	37	-18	3.99
Frontal_Mid	R	89	25	29	40	4.37

R		30	39	34	4.33

Note. Thresholded at $p < .001$, uncorrected; minimum of 20 voxels; [1]cluster contains ventral striatum; [2]cluster contains parahippocampal cortex; [3]cluster contains hippocampus; [4]cluster contains paracingulate cortex including the retrosplenial cortex; [5]cluster contains ventromedial prefrontal cortex.

Table S4. Results from small volume correction within the ventral striatum ROI.

p(FWE-corr)	p(unc)	t-value	x	y	z
< 0.001	< 0.001	8.49	6	12	-7
< 0.001	< 0.001	7.68	-4	7	-7
< 0.001	< 0.001	7.56	-7	14	-7
< 0.001	< 0.001	7.30	-4	12	-4
0.006	< 0.001	4.33	-14	9	-12

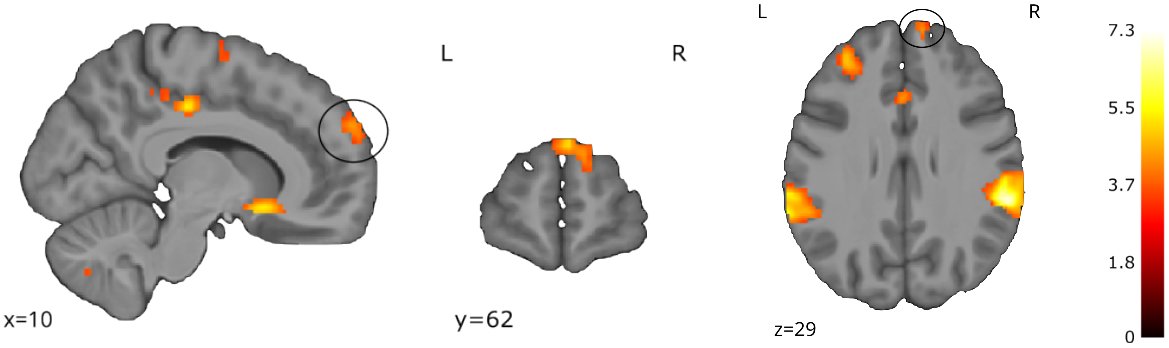

Figure S8. Choice value (Q) parametric modulation analysis (n = 49). The slices show the dmPFC cluster. For illustration purposes, the *t*-map was thresholded at $p < .001$ (uncorrected) with a minimum cluster size of 20 voxels and overlaid on the mean T1 image.

Table S5. Choice value (Q) modulates univariate whole-brain activity.

AAL Label	Hemisphere	Voxels	x	y	z	*t*-value
Cerebelum_Crus2	L	319	-24	-75	-40	7.36
	L		-34	-85	-32	5.39
	L		-22	-85	-37	5.01
SupraMarginal	R	2310	60	-35	29	7.11
	R		58	-26	26	6.5
Putamen	R		30	-18	4	6.41
Cerebelum_Crus2	R	214	20	-75	-37	6.85
	R		33	-85	-34	5.01
Temporal_Mid	L	117	-54	2	-15	5.99
Temporal_Pole_Sup	L		-42	2	-15	3.59
Cingulum_Mid	R	452	8	-21	42	5.89
Precuneus	L		-7	-53	59	5.12
Postcentral	L		-24	-43	67	5.05
Insula	L	844	-39	7	1	5.93
Putamen	L		-27	4	10	5.58
	L		-27	-8	4	5.47
Cingulum_Mid	R	466	8	-21	42	5.92
Precuneus	L		-7	-53	59	5.18
Postcentral	L		-24	-43	67	5.11
SupraMarginal	L	334	-67	-43	29	5.67
	L		-62	-40	37	5.59
	L		-67	-33	26	5.54
Frontal_Mid	L	147	-32	47	26	5.35
	L		-24	39	26	4.58
	L		-32	32	45	3.91
Frontal_Sup_Medial *	R	111	3	62	34	5.21

	L		-4	52	48	4.6
Cerebelum_8	L	38	-22	-65	-54	4.8
Temporal_Mid	L	83	-62	-55	-2	4.69
	L		-57	-63	-2	4.2
	L		-54	-55	10	3.81
Cingulum_Ant	R	27	1	24	29	4.31
Supp_Motor_Area	R	93	6	7	70	4.18
	R		8	-3	64	3.85
	R		6	24	64	3.79
Temporal_Sup	L	26	-57	-28	10	4.18
Frontal_Mid	R	22	28	54	23	4.02
Frontal_Sup	L	27	-19	-1	70	3.66
Supp_Motor_Area	L		-9	-8	70	3.59

Note. Thresholded at $p < .001$, uncorrected; minimum of 20 voxels. *cluster contains dmPFC ROI.

Table S6. Results from small volume correction within the dmPFC ROI.

p(FWE-corr)	p(unc)	t-value	x	y	z
0.002	< 0.001	4.33	10	62	29

Supplementary Note 4

Searchlight representational similarity analysis (RSA) results

We used searchlight RSA analysis to identify brain regions that encode representations of individual people (sphere radius of 7.5 mm, 3 voxels). We used functions from the RSA toolbox [8] and compared activity patterns across runs in a similar fashion as done for the ROI-based RSA. Before running the second-level analysis, first-level maps were z-transformed, normalized to MNI space using the fMRIprep transform files, and smoothed with a Gaussian Kernel of 6 mm radius of FWHM.

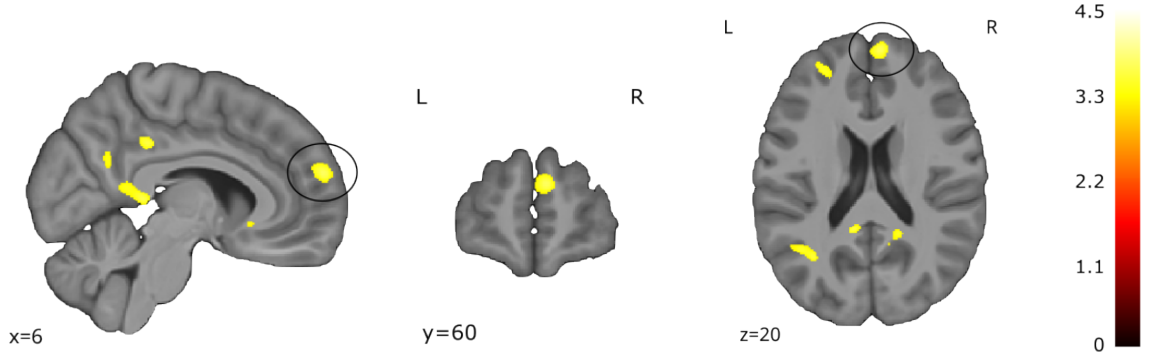

Figure S9. Searchlight RSA results (n = 49). The slices illustrate the dmPFC cluster. For illustration purposes, the *t*-map was thresholded at $p < .001$ (uncorrected) with a minimum cluster size of 20 voxels and overlaid on the mean T1.

Table S7. Results of the searchlight representational similarity analysis.

AAL Label	Hemisphere	Voxels	x	y	z	*t*-value
Occipital_Sup	L	10119	-22	-73	37	4.47
	L		-26	-68	31	4.25
	L		-29	-86	30	4.13
Temporal_Pole_Mid	R	1660	41	11	-34	4.46
Temporal_Mid	L	4706	-42	-54	14	4.29
	L		-51	-56	9	4.2
	L		-47	-49	2	3.72
Cingulum_Mid	L	2245	-5	-25	39	4.21
	R		4	-35	37	3.8
	L		-9	-22	47	3.67
Occipital_Mid	R	2140	38	-78	26	4.18
Frontal_Sup	L	1346	-19	38	40	4.1
Frontal_Mid	L		-28	31	49	3.48

Precuneus	R	1917	16	-47	13	4.08
Cingulum_Post	R		6	-39	7	3.69
Precuneus	R	1098	16	-58	35	4.08
	R		27	-56	28	3.46
Fusiform	R	466	39	-37	-13	4.05
Fusiform	L	1757	-25	-41	-17	4.02
	L		-34	-30	-21	3.38
Cingulum_Post	L	2071	-9	-45	24	4.01
Precuneus	L		0	-57	32	3.95
Putamen	R	300	28	0	9	3.97
Caudate	L	273	-27	-11	32	3.92
Putamen	L	313	-29	3	1	3.91
Cingulum_Ant	L	830	-16	43	12	3.87
	L		-8	41	12	3.57
Cerebelum_10	L	214	-11	-29	-39	3.79
Frontal_Sup_Medial *	R	643	6	60	21	3.75
Precentral	L	83	-29	-10	75	3.68
Frontal_Sup_Orb	R	236	21	23	-11	3.64
Thalamus	L	79	-19	-19	4	3.63
Hippocampus	L	145	-13	-36	4	3.61
Hippocampus	R	205	39	-17	-10	3.57
Frontal_Med_Orb	L	129	-1	52	-8	3.53
Frontal_Mid	L	290	-28	49	22	3.53
Precuneus	L	160	-2	-52	57	3.52
Frontal_Sup	L	292	-18	23	60	3.5
Frontal_Inf_Orb	R	47	42	39	-8	3.47
Putamen	R	42	30	15	-2	3.42
Frontal_Sup_Medial	L	176	-9	51	32	3.4
Olfactory	R	22	6	20	-7	3.38

Note. Thresholded at $p < .001$, uncorrected; minimum of 20 voxels. *cluster contains dmPFC ROI.

Table S8. Results from small volume correction within the dmPFC ROI.

p(FWE-corr)	p(unc)	*t*-value	x	y	z
0.007	< 0.001	3.73	6	60	20
0.019	0.001	3.33	7	65	21

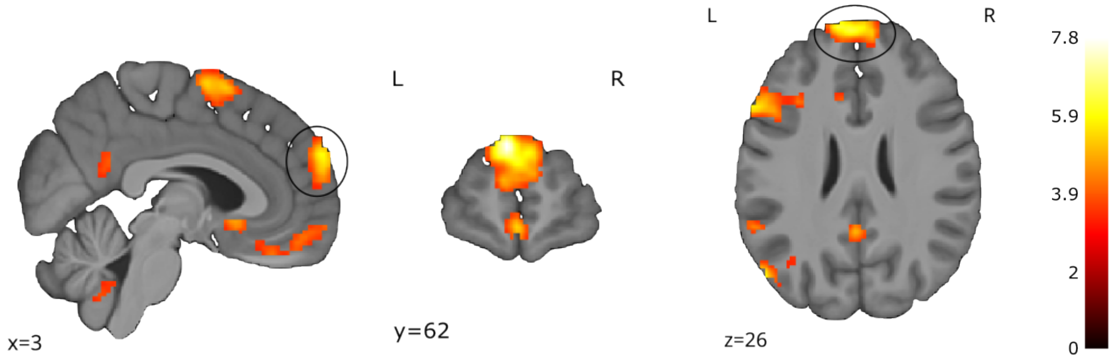

Figure S10. PPI analysis (n = 49). The slices illustrate the dmPFC cluster. For illustration purposes, the *t*-map was thresholded at $p < .001$ (uncorrected) with a minimum cluster size of 20 voxels and overlaid on the mean T1.

Table S9. Brain regions exhibiting PE-dependent coupling with the ventral striatum.

AAL Label	Hemisphere	Voxels	x	y	z	*t*-value
Frontal_Sup_Medial *	L	650	-7	62	32	7.91
	R		3	62	29	5.92
	L		-9	59	15	5.23
Temporal_Pole_Sup	L	1877	-39	22	-29	7.2
Frontal_Inf_Orb	L		-47	32	-2	7.03
Frontal_Inf_Tri	L		-59	24	10	6.61
Temporal_Pole_Sup	R	423	48	22	-26	7.19
Temporal_Sup	R		58	4	-12	5.87
Frontal_Inf_Orb	R		38	39	-7	5.68
Supp_Motor_Area	L	736	-4	12	54	7.06
	L		-2	2	64	6.09
	R		6	12	64	5.53
Frontal_Mid	L	314	-42	4	51	6.51
Precentral	L		-52	-1	48	4.74
Frontal_Sup	L		-24	-3	56	4.41
Occipital_Inf	L	732	-44	-73	-15	6.5
Calcarine	L		-12	-103	-10	5.99
Lingual	L		-32	-95	-15	5.91
Calcarine	R	502	18	-100	-4	6.34
Occipital_Inf	R		33	-95	-4	5.58
Lingual	R		23	-90	-12	5.45
Precuneus	L	371	-4	-55	10	6.2
Calcarine	L		-14	-48	7	5.91
Precuneus	L		-9	-58	20	5.09
Frontal_Inf_Orb	R	118	53	32	-2	6.07

Olfactory	R	445	1	17	-7	6.06
Rectus	L		-2	49	-21	5.8
Frontal_Med_Orb	L		-2	64	-10	5.78
Hippocampus	R	100	25	-13	-12	5.73
Fusiform	R		25	-28	-18	4.65
Hippocampus	R		18	-21	-10	3.78
Angular	L	133	-52	-75	26	5.68
Occipital_Mid	L		-39	-80	37	5.4
	L		-37	-65	26	3.45
Hippocampus	L	128	-24	-16	-12	5.3
	L		-34	-11	-15	5.05
	L		-19	-6	-15	4.67
Cerebelum_9	R	64	8	-48	-46	5.2
Temporal_Mid	L	27	-57	-48	23	4.92
Caudate	L	44	-14	12	12	4.6
Pallidum	L		-14	4	-4	3.6
Putamen	L		-19	9	1	3.51
Precentral	R	51	38	-23	51	4.47
	R		30	-28	59	3.79
Cerebelum_Crus2	R	46	15	-83	-43	4.44
Cerebelum_8	R	36	35	-60	-54	4.33
	R		25	-68	-51	4.02
Cerebelum_Crus1	R	45	38	-60	-34	4.11
	R		35	-63	-26	3.97
Temporal_Sup	R	23	48	-23	1	3.89
	R		43	-33	4	3.73
	R		53	-30	4	3.62

Note. Thresholded at $p < .001$, uncorrected; minimum of 20 voxels. *cluster contains dmPFC ROI.

Table S10. Results from small volume correction within the dmPFC ROI.

p(FWE-corr)	p(unc)	t-value	x	y	z
< 0.001	< 0.001	5.61	3	62	26
< 0.001	< 0.001	5.24	3	67	26
0.030	0.001	3.4	6	57	15

Supplementary References

1. Wilson, R. C. & Collins, A. G. Ten simple rules for the computational modeling of behavioral data. *eLife* **8**, e49547 (2019).
2. Pruessner, J. C., Kirschbaum, C., Meinlschmid, G. & Hellhammer, D. H. Two formulas for computation of the area under the curve represent measures of total hormone concentration versus time-dependent change. *Psychoneuroendocrinology* **28**, 916–931 (2003).
3. Garcia Jimenez, C., Mazzoni, G. & D'Argembeau, A. Repeated simulation increases belief in the future occurrence of uncertain events. *Mem. Cognit.* **51**, 1593–1606 (2023).
4. Szpunar, K. K. & Schacter, D. L. Get real: Effects of repeated simulation and emotion on the perceived plausibility of future experiences. *J. Exp. Psychol. Gen.* **142**, 323–327 (2013).
5. Lee, S. *et al.* A neural signature of the vividness of prospective thought is modulated by temporal proximity during intertemporal decision making. *Proc. Natl. Acad. Sci.* **119**, e2214072119 (2022).
6. Soch, J. & Allefeld, C. MACS - a new SPM toolbox for model assessment, comparison and selection. *J. Neurosci. Methods* **306**, 19–31 (2018).
7. Daunizeau, J., Adam, V. & Rigoux, L. VBA: a probabilistic treatment of nonlinear models for neurobiological and behavioural data. *PLoS Comput. Biol.* **10**, e1003441 (2014).
8. Nili, H. *et al.* A Toolbox for Representational Similarity Analysis. *PLOS Comput. Biol.* **10**, e1003553 (2014).
